# Reducing the failure rate of hip resurfacing in dysplasia patients: a retrospective analysis of 363 cases

**DOI:** 10.1186/s12891-016-1095-7

**Published:** 2016-06-07

**Authors:** Melissa D. Gaillard, Thomas P. Gross

**Affiliations:** Midlands Orthopaedics & Neurosurgery PA, 1910 Blanding Street, Columbia, SC USA

**Keywords:** Hip resurfacing, Hip dysplasia, Hip arthroplasty, Hip replacement, Metal-on-metal, Adverse wear

## Abstract

**Background:**

Arthritis secondary to developmental hip dysplasia often mandates implant surgery at a relatively young age. Hip resurfacing arthroplasty (HRA), compared with standard stemmed total hip arthroplasty (THA), affords a more active lifestyle including extreme-motion activities but stimulates concerns pertaining to implant failure.

**Methods:**

We addressed the primary modes of failure through a series of interventions, including a new guideline for achieving proper implant alignment through intraoperative x-rays. We then compared two sequential cohorts in a single-surgeon practice: patients with developmental dysplasia who underwent HRA before (Group 1; 121 hips in 105 patients) and after (Group 2; 242 hips in 210 patients) June 2008, at which time the four interventions were all in place.

**Results:**

Implants in Group 2 failed less frequently within two years (0.8 % vs. 6.6 %, *p* = 0.002) and were more likely to have projected seven-year Kaplan-Meier survivorship (99 % vs. 89 %, *p* < 0.0001 by log-rank test). Patients in Group 2 were more likely to have normal metal ion levels (77 % vs. 56 %, *p* = 0.0008) and optimum metal ion levels (99 % vs. 86 %, *p* = 0.0008). Patients in Group 2 also benefited from a 19-min decrease in mean operation time, a 45 % decrease in mean estimated blood loss, and a 0.9-day decrease in mean hospital stay (*p* < 0.0001 in each instance).

**Conclusions:**

We believe the interventions reported here, combined with sufficient surgeon experience and properly designed implants, afford patients with mild developmental dysplasia a more active lifestyle with favorable implant survival.

## Background

Deformities of the acetabulum and femur make arthritis due to developmental hip dysplasia a unique challenge for orthopedic surgeons. The shallow, oval-shaped acetabulum complicates proper implant alignment. Increased tissue laxity motivates many patients with hip dysplasia to self-elect high range-of-motion (ROM) activities such as ballet and gymnastics, which then accelerate the development of arthritis and increase the likelihood of prosthetic instability [1, 2] (See List of Abbreviations). Theoretical advantages of hip resurfacing arthroplasty (HRA) over standard stemmed total hip arthroplasty (THA) for these patients include greater hip stability [3–5], less thigh pain [6, 7], more nearly normal gait [8–10], and resumption of high ROM activities [11–13]. However, reported failure rates for both HRA and THA run higher for patients with dysplasia than in most other patient populations. Failure rates have been especially high in women who are more likely than men to have this disorder [11, 14, 15].

We have long suspected that the technical difficulties of achieving optimum alignment with HRA explain at least in part the divergent results from registry data. The Australian joint registry, perhaps the most comprehensive project of its kind, indicates that women under age 60 experience higher failure rates with HRA than with THA, while the reverse holds for men [14, 16, 17]. On the basis of these and similar data, many professionals advise against HRA for younger women. Paradoxically, experts do not advise against THA for younger men despite their reported lower failure rates with HRA. The greater technical difficulty of HRA compared to THA possibly explains this inconsistency. With THA, amputation of the femoral neck allows the surgeon to achieve better exposure while with HRA, preservation of the femoral head makes acetabular preparation a technical challenge and a barrier to better results and wider acceptance of this procedure [18, 19].

We have sought to identify principal causes of failure and to develop strategies to eliminate each failure mode. In patients with development dysplasia, we previously determined [20] the main causes of revision to be the failure of acetabular ingrowth (FAI) and adverse wear related failure (AWRF). In contrast to other reports [1, 9], we did not find early femoral failure (EFF) to be more common in dysplasia patients. We therefore focused mainly on developing strategies to prevent FAI and AWRF, while not ignoring EFF. We also developed an uncemented femoral component with the aim of reducing the 3 % rate of late loosening of the cemented femoral component that occurred in our resurfacing cases [3, 21].

In this paper, we describe four specific strategies (interventions) and their combined impact on outcomes of HRA for arthritis secondary to developmental dysplasia of the hip. These consist of *improved acetabular component fixation* to address FAI, including use of the Magnum™ Tri-Spike component in higher-risk cases with the largest deformities; *improved acetabular component alignment,* intended to prevent AWRF*,* through a better understanding of optimum positions and ways to obtain them, notably use of a normalized intraoperative x-ray technique as well as use of a new guideline for component alignment (see Materials and Methods); a *bone protection program,* designed to prevent EFF*,* based on modified early weight-bearing and alendronate with emphasis on individual patients’ bone density femoral neck T-scores and body mass indices (BMI); and *uncemented femoral fixation*, intended to reduce the rates of EFF and early femoral loosening. We analyzed the impact of these strategies by comparing two sequential cohorts of patients undergoing HRA for developmental dysplasia: one before, and one after, all of these interventions were in place.

## Methods

### Patients and follow-up

The senior author has maintained a prospective database of more than 4200 HRA procedures, of which 518 (12 %) have been in patients with underlying dysplasia. Procedures were performed at Providence Northeast Hospital, Lexington Medical Center, and Midlands Orthopaedics Surgery Center, all located in Columbia, SC. The database has a 96 % overall follow-up rate. The present analysis includes 121 HRA procedures for dysplasia performed between January 2001 and July 2008 (Group 1) and 242 procedures performed between August 2008 and July 2013 (Group 2), all with minimum 2-year follow-up. Approvals for this study and report were obtained from the Institutional Review Board of Sisters of Charity Providence Hospitals, Columbia, South Carolina.

Patients are born with varying amounts of ovoid deformity and erosion gradually increases their dysplasia grade. Degree of dysplasia was graded as follows: *Grade 1*, 50 % to 80 % coverage of the femoral head on standing anterior posterior (AP) x-ray, with less than 1 cm of superior migration; *Grade 2*, less than 50 % coverage, but less than 2 cm of superior migration; *Grade 3*, more than 2 cm of superior migration of the head, irrespective of coverage; *Grade 4*, superior migration with a false acetabulum; and *Dysplasia with Osteotomy*, deformity by previous femoral and/or acetabular osteotomy. Osteophytes are excluded when judging coverage. Most patients with dysplasia inexorably progress to higher grades as relentless bone wear elongates the socket and subluxes the femoral head into the superior aspect of the oval defect, from which it eventually migrates out of the socket. We characterize socket anteversion as an oval acetabulum. Preoperative AP pelvic x-rays demonstrate variable amounts of head coverage and superior migration. Intraoperatively, all patients are found to have oval sockets (osteoarthritic sockets with eccentric bone wear excluded).

#### Implant systems

The senior author used three separate implant systems in a consecutive fashion. Beginning in March 2001, Hybrid Corin Cormet 2000 (Corin, Cirencester, UK) devices were employed as part of a multicenter United States Food and Drug Administration trial. Beginning in 2005, Biomet (Biomet, Warsaw, Indiana) devices comprised of a cemented ReCap™ femoral component and an uncemented Magnum™ acetabular component were used. Beginning in 2007, we initiated the use of completely uncemented Biomet ReCap™ Magnum™ devices, with the Magnum™ Tri-Spike component used in higher-risk patients with the largest deformities. Currently, all devices are uncemented. Using these Biomet devices in the procedure reported here falls under the category of off-label use in the United States.

#### Procedure

This study spans a period of 11 years during which surgical technique evolved. A summary of surgical information from these cases is listed in Table 1. Over time, we recognized several key features in dysplasia patients: The acetabulum is always oval to a varying degree, which is present at birth and increases as the hip wears. The long axis of the oval is *always* from posterior inferior to anterior superior. The head rides into the superior corner of the oval and then begins to sublux. The thickest bone for placing the cup is posterior inferior. The cup is shallow, and medial wall thickness is variable (Fig. 3a). These patients exhibit hypermobility and are prone to dynamic posterior pelvic tilt on standing. The transverse acetabular ligament (TAL) is an excellent reference point for judging correct anteversion.

**Table 1:** Demographics

	Pre-2008, Group 1 (*N* = 121)	Post-2008, Group 2 (*N* = 242)	*P*-value
*Sex (no. of hips)*			
MALE	35 (29 %)	64 (26 %)	0.6171
FEMALE	86 (71 %)	178 (74 %)	
DECEASED^a^	3 (2.5 %)	1 (0.4 %)	0.0751
* Follow-up Mean Years*	6.4 ± 2.31	2.6 ± 1.51	<0.0001^b^
* Case Date Range*	1/2001–7/2008	7/2008–7/2013	--
* Age (yr)*	48 ± 8.37	52 ± 7.05	<0.0001^b^
* BMI*	26 ± 5.04	26 ± 5.08	1.000
* T-score*	−0.34 ± 1.44	−0.40 ± 1.10	0.6599
* Overall Failures (no. of hips)*	16 (13.2 %)	2 (0.8 %)	<0.0001^b^
* 2-Year Raw Failures (no. of hips)*	8 (6.6 %)	2 (0.8 %)	0.0015^b^
* 7-Year Survivorship (no. of hips)*	108 (89.3 %)	240 (99.2 %)	<0.0001^b^
*Dysplasia Grade (no. of hips)*			
Dysplasia I	37/51 (73 %)	188/241 (78 %)	0.4009
Dysplasia II	10/51 (20 %)	49/241 (20 %)	0.9045
Dysplasia III	0/51 (0 %)	0/241 (0 %)	1.000
Dysplasia IV	0/51 (0 %)	0/241 (0 %)	1.000
Dysplasia Osteotomy	4/51 (7.8 %)	4/241 (1.7 %)	0.0139^b^
Grade Unrecorded	70/121 (58 %)	1/242 (0.4 %)	<0.0001^b^
* ASA Score*	1.6 ± 0.55	1.6 ± 0.56	1.000
* Femoral Component <48 mm (no. of hips)*	46/121 (38 %)	79/242 (33 %)	0.3077
* Femoral Component Size*	48.1 ± 3.45	47.0 ± 2.91	0.0016^b^

^a^Died with the causes unrelated to their hip arthroplasties

^b^Statistically significant

**Fig. 1:**
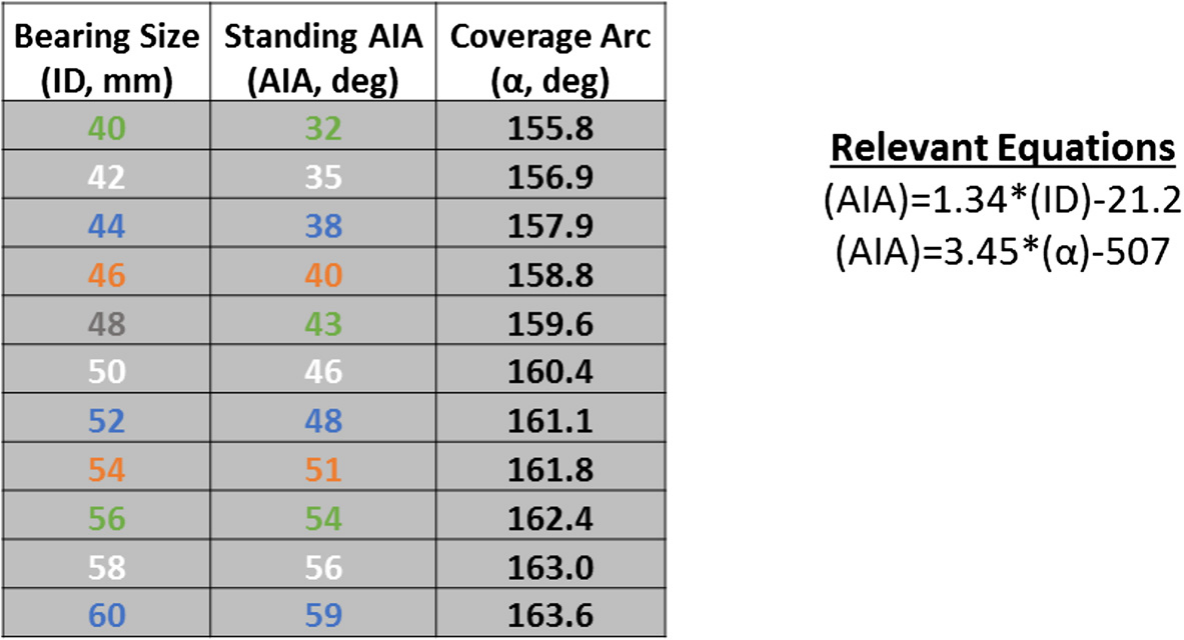
RAIL Reference Table. RAIL guideline for correct acetabular component placement to avoid high ion levels and AWRF in HRA. The lower limit is arbitrarily set at 25°, the upper limit of acceptable AIA is listed for each bearing size. For the smallest bearing size (40 mm) there is only a 7° window, making placement very challenging

**Fig. 2:**
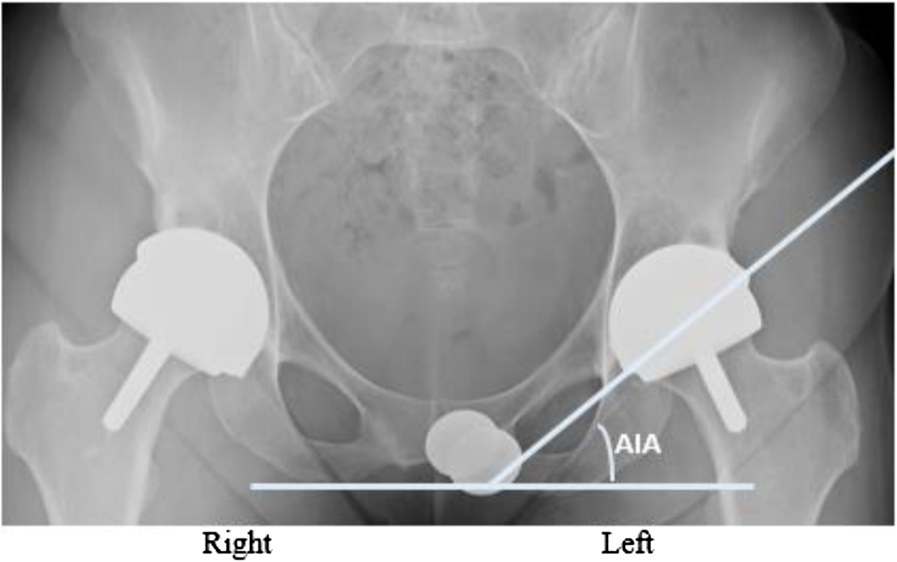
Radiograph Measurement References. Anterior-posterior pelvis radiograph made five years after a hybrid metal-on-metal Corin hip resurfacing on the right hip and two years after a hybrid metal-on-metal Biomet ReCap™ hip resurfacing on the left hip. Better AIA is noted in the most recent HRA on the patients left side

**Fig. 3:**
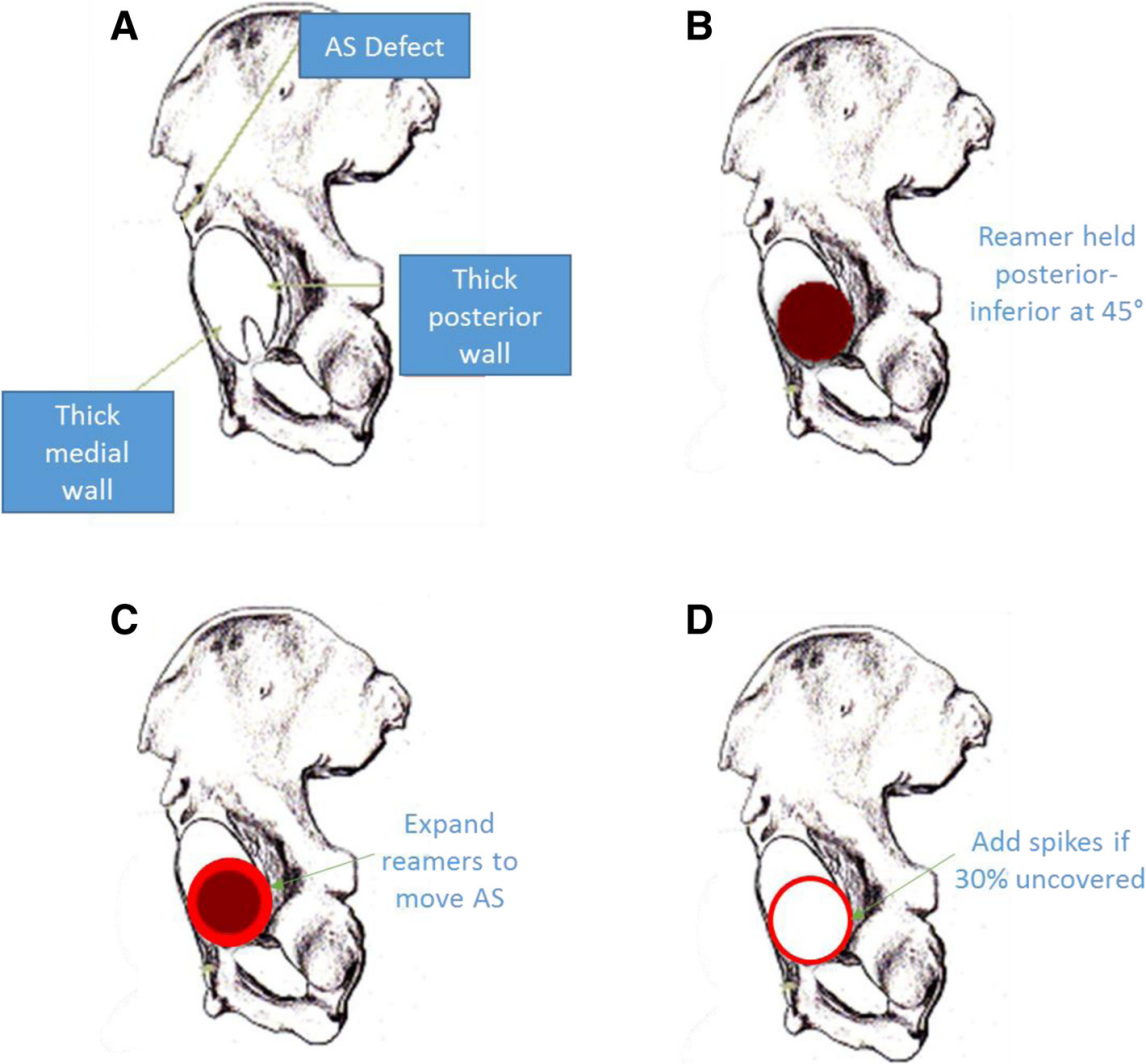
Dysplasia technique. **a** The typical oval dysplasia deformity is illustrated. **b** Initial reaming is demonstrated. **c** Reamers are gradually expanded to capture oval defect. **d** Cup stability is judged by amount of trial that remains uncovered anterior-superiorly

DeSmet [22] demonstrated that steeper, more anteverted components were problematic especially in dysplasia patients with smaller bearing sizes (34 % patients in this study had bearing size < 48 mm). He defined the useful concept of *coverage arc*, which depends largely on the design features of the implant and its proper surgical positioning. We further developed this concept with the Relative Acetabular Inclination Limit (RAIL) guideline [23] (Fig. 1) for placing components based on bearing size. The RAIL dictates an acetabular inclination angle (AIA) limit on a *standing AP pelvis x-ray* that is different for each bearing size*.* We placed an arbitrary lower AIA limit of 25° on the guideline. We next developed a technique to obtain an intraoperative x-ray that was normalized to the standing position (unpublished). These techniques were fully established by mid-2008.

All operations were performed by a single surgeon (TPG). Cemented femoral components were implanted using small amounts of high-viscosity cement on both the bone and implant surfaces with an escape trough machined into the femoral head. In Group 2, stems were never cemented.

The posterior approach used in these cases was described previously [24]. A 4- to 6- in. skin incision is made; the gluteus maximus, quadratus femoris, and all 3 short rotators are released off of the bone and are subsequently repaired, the conjoined tendon is incised 1–2 cm, a neck-sparing *complete* capsular division and repair is performed. A pocket is created under the minimus for later placement of the head.

The head is first prepared. Typically, it is oval and foreshortened. Therefore, more is typically trimmed from the periphery than from the apex. The neck gauge is applied to measure the smallest femoral component possible. The central guidewire is placed. Although it is not critical, we attempt to place the stem neutral down the center of the neck and parallel to the calcar. There is no way to alter femoral version, and we are of the opinion that it does not matter. We have never found the need for a femoral derotation osteotomy. The head is then typically cut 6 mm (3 sizes) larger than the smallest component possible (neck gauge). If there is doubt, we cut one size larger (2 mm) and later come back and trim it down if necessary after the acetabulum is implanted. The trial head is placed and the head is then tucked into the pocket under the minimus and held with a double-angled Hohmann retractor.

**Fig. 4:**
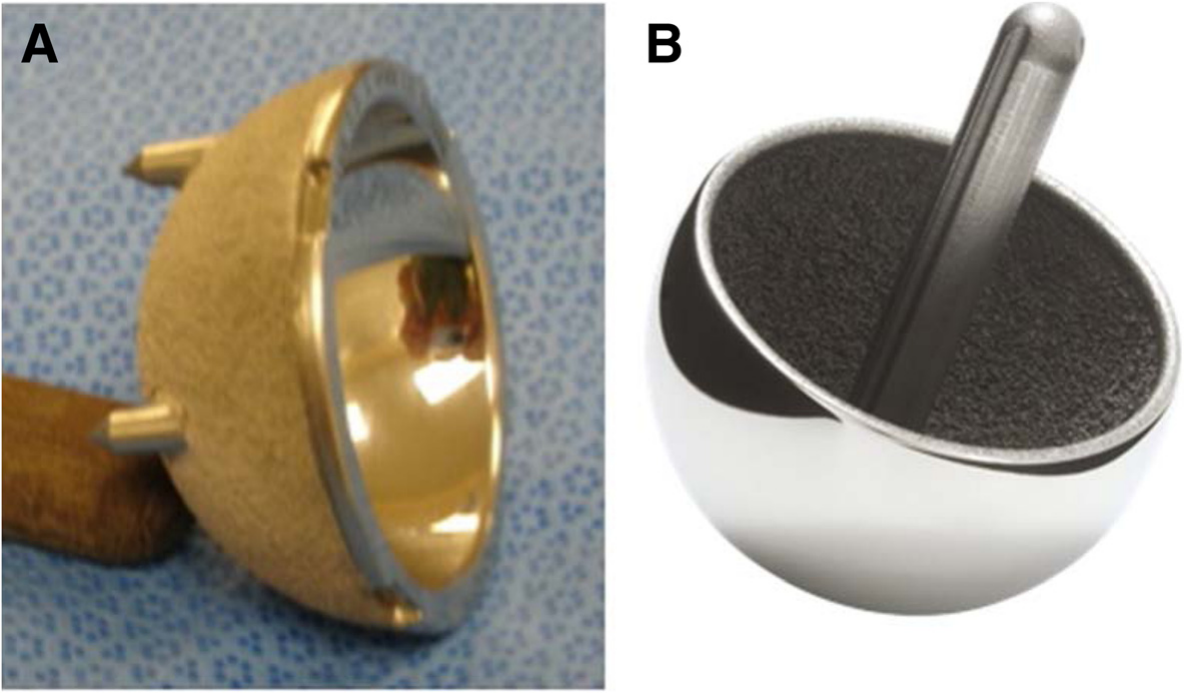
ReCap™ Components. **a** Tri-Spike Magnum™ component used if less than 70 % cup coverage is achieved. **b** Uncemented Biomet ReCap™ femoral component

The thickened labrum is excised except for a small band in the anterior inferior region against the psoas. The TAL must be preserved during this maneuver, because it is the best guide for anteversion. We find it a reliable guide even in the face of the acetabular deformity. The reamer equal to the prepared head size is used first. It is inserted at a 45° angle with the floor but *pulled firmly* inferiorly and posteriorly while pushing centrally (Fig. 3b). It is *forced* to progress into the thick posterior wall and down to the quadrilateral plate. If the surgeon is not careful and allows the reamer to seat anterior superior (AS) into the oval defect while reaming, the entire preparation is incorrect and cannot be salvaged. This cannot be overemphasized. This initial preparation serves several purposes. First, the cavity is prepared in the thicker posterior bone. Second, the anterior inferior (AI) bone adjacent to the psoas is preserved. Third, the center of rotation is corrected in high-riding cases. The medial wall is then drilled at the superior edge of the fovea and a depth gauge is used to measure the medial wall. For proper cup coverage, the cavity can be medialized until 6 mm remains medially. As soon as the optimum medial position has been achieved, larger reamers are used to expand the cavity (Fig. 3c). The reamer handle is still firmly held posteriorly and inferiorly, but the grip is slightly loosened to allow the reamer to progress slightly into the oval defect AS. A line-to-line reaming is performed if the dual energy x-ray absorptiometry (DEXA) T-score exceeds −1.0 (good bone). A 1-mm under-reaming is employed when the T-score falls below −1.0 (soft bone) (T-score is a reference to gender and race specific bone density in a healthy, young 25-year old [25]; a normal T-score is from 1 to −1). A clean reamer 1 mm larger than the head size is then used to deepen the apex of the cavity by 1–2 mm. This prevents the cup from bottoming out at the apex of the cavity and instead creates a wedge fit on the rim. The trial is placed and aligned with the TAL with an AIA dictated by the RAIL guideline. If a significant amount of the oval defect remains such that 30 % or more of the component wall is uncovered (Fig. 3d), a Tri-Spike component (Fig. 4) is chosen. If the preoperative T-score is less than −2.5, a Tri-Spike is also chosen. Otherwise, a standard Magnum™ cup is used. Approximately 5 % of these cases required a Tri-Spike component since it became available. The trial is used to determine if three criteria are met: AIA is within RAIL guidelines, anteversion is parallel to the TAL, and the cup edge is below the bone edge in the AI corner adjacent to the psoas. If these criteria are not met, the cavity typically needs to be deepened slightly to allow the trial to satisfy these conditions. After assuring the trial criteria are met, the cup, which is hemispherical with a rough titanium plasma spray coating and 4 sharp fins, is lightly impacted into the prepared cavity with a curved inserter. The position is then finely tuned with a plastic tip tamp and mallet. If the cup needs to be removed, a metal tip tamp is used on the exposed edge. When the position is correct, a secondary impactor designed to ride over the edge of the cup is used for a final impaction with a large mallet. If a Tri-Spike component is used, repositioning requires removal; technically, these are more difficult to orient perfectly according to the above criteria.

The head is trimmed if necessary to match the acetabular component. The Magnum™ cup outer diameter is 6 mm larger than the head/bearing size. All cysts are curetted and grafted. The ReCap™ component is impacted until sonic change indicates that it is fully seated. The hip is reduced and an x-ray is obtained. Closure is routine, repairing all structures anatomically in multiple layers.

#### Normalized to standing intraoperative x-ray

The normalized intraoperative x-ray technique (fully-developed by mid-2008) utilizes a portable, digital x-ray machine that enables immediate comparison of an image taken in the operating room (OR) with the patient’s preoperative standing x-ray. After the acetabular component is implanted according to the technique described previously [24], the OR table is rotated until the surgeon judges the pelvis to be neutral. The first film is taken. The surgeon inspects the width of the obturator foramina on the image and then asks the anesthetist to roll the table right or left to correct for any rotation. Films are repeated until the widths of the obturator foramina are symmetrical. The surgeon then compares the height of the obturator foramina to the height seen on the preoperative *standing AP pelvis x-ray.* The x-ray machine is tilted cephalad or caudad and films are repeated until the obturator foramina and the pelvic inlet appear as they do on the preoperative standing AP pelvis x-ray. Thus, the end result is an intraoperative image of the pelvis *normalized* to the patient’s preoperative standing position. This image is sent to the desktop computer in the OR and the AIA is measured. If the AIA does not meet RAIL criteria (Fig. 4), the cup is repositioned and another x-ray is obtained. With practice, this process typically takes less than 2 min, and less than 5 % of cups need repositioning.

#### Postoperative protocol

We previously concluded that the risk of EFF (early femoral failure: femoral neck fracture and collapse) depends largely on two factors: bone density (femoral neck T-score) and BMI [26]. Our bone management protocol establishes three groups based on these risk factors: Group A, femoral neck T-score score > 0 and BMI < 30; Group B, femoral neck T-score between 0 and −1.5 and/or BMI > 30; and Group C, femoral neck T-score < −1.5.

Group A patients are allowed to progress weight-bearing as tolerated (WBAT). They typically use crutches for 1–2 weeks and a cane for 1–2 weeks. Group B patients are also allowed WBAT but are prescribed alendronate for 6 months. Patients from Group C or any patients who require a Tri-Spike cup are placed on a slowed weight-bearing protocol and are prescribed alendronate for 1 year. They are asked to do 10 % weight-bearing for 4 weeks, WBAT with crutches for 2 weeks, and a cane for another 4 weeks. Isometric leg lifts, light aerobic activity (such as exercise bike and swimming), lower extremity weight lifting less than 50 lb, golf, and unrestricted walking is encouraged at 6 weeks for Groups A and B and at 10 weeks for Group C patients. All restrictions in all patients are lifted at 6 months, and they are encouraged to participate in full-impact sports. No formal physical therapy is requested of the patient after hospital discharge.

Deep vein thrombosis precautions include sequential compression devices started intraoperatively and discontinued at the time of hospital discharge. Ambulation and leg exercises are started within 24 h. Oral anticoagulants are started at 24 h postoperative and continued for 2–4 weeks, depending on risk analysis. Then, 81 mg aspirin is recommended for another month.

A multimodal pain management protocol and a comprehensive blood management protocol are used to eliminate transfusion and speed recovery. In the last 3 years, resurfacing has been performed as an outpatient procedure on selected patients.

#### Metal ion testing

Our database contains blood, serum, and plasma ion levels for these cases. We used the method of Smolders [27, 28] to convert all serum and plasma levels to whole blood levels, and thus, we used blood levels for all comparisons. DeSmet and colleagues [22] demonstrated that blood ion levels provide an excellent surrogate marker, or screening tool, for wear and are thus useful for screening for AWRF prior to the onset of any symptoms. We define 5 different ion level categories based on previous research [22, 23, 27, 28]: normal, optimal, acceptable, problematic, and potentially toxic. These reference ion values are shown in Table 2.

**Table 2:** Metal ion reference values

	Normal^a^	Optimal^b^	Acceptable^c^	Problematic^c^	Potentially Toxic^b^
Unilateral					
• Co	<1.5 μg/L	<4.0 μg/L	4–10 μg/L	10–20 μg/L	>20 μg/L
• Cr	<1.5 μg/L	<4.6 μg/L	4.6–10 μg/L	10–20 μg/L	>20 μg/L
Bilateral					
• Co	<1.5 μg/L	<5.0 μg/L	5–10 μg/L	10–20 μg/L	>20 μg/L
• Cr	<1.5 μg/L	<7.4 μg/L	7.4–10 μg/L	10–20 μg/L	>20 μg/L

^a^laboratory normal for patients without metal bearings

^b^according to DeSmet /van der Straeten

^c^according to our previous analysis

#### Clinical and radiological analysis

Office or remote follow-up was requested at 6 weeks, 1 and 2 years, and every other year thereafter. A clinical questionnaire, radiographs, and a physical examination testing ROM and strength were performed at each visit. After 1 year, physical examinations were no longer done routinely on remote follow-ups. The OrthoTrack database (Midlands Orthopaedics, Columbia, South Carolina) supported our collection and analysis of the demographic, clinical, and radiographic data for all patients.

Patient questionnaires requested information necessary to calculate the following scores for clinical evaluation: Harris hip score (HHS) [29], University of California, Los Angeles (UCLA) activity score [30], and visual analog scale (VAS) pain score for normal and worst days [31]. HHS determines clinical outcome; UCLA activity scores measure activity level after surgery on a scale from 1 to 10, for which 10 represented the highest level of activity; VAS pain scores rate the level of pain from 0 to 10, with zero representing no pain and 10 representing maximum levels of debilitating pain.

Both supine and standing AP pelvis and lateral radiographs are taken and analyzed for component position, shifting, and radiolucencies by the senior author. The AIA is determined by measuring the angle formed between 2 straight lines: one running across the face of the acetabular component and the other across the inferior pubic rami (Fig. 2). All measurements were performed using OrthoTrack (Midlands Orthopaedics).

#### Statistical methods

The significance level α was defined as 0.05 for all statistical analyses in this study. A paired, 2-tailed *T*-test was used to calculate the significant difference between preoperative and postoperative numerical outcomes within and between dysplasia study groups; the Student’s *T*-test was used to compare the difference of numeric variables between groups. When comparing two population proportions, a two-sample Z-test was used. Statistical tests were performed using SAS (Cary, NC). Kaplan-Meier survivorship curves were plotted to evaluate implant survival among different groups. Log-rank and Wilcoxon tests were performed to calculate significant differences between survivorship curves. Curves and survivorship statistical tests were generated using XLSTAT (New York, NY).

## Results

Groups 1 and 2 were demographically similar, although patients enrolled subsequent to mid-2008 (Group 2) tended to be slightly older and less likely to have had dysplasia with a prior osteotomy (Table 1). Patients in Group 2 had significantly shorter operation times (mean 95 min versus 114 min in Group 1, *p* < 0.0001), significantly less estimated blood loss (mean 142 mL versus 257 mL in Group 1, *p* < 0.0001), and significantly shorter hospital stays (mean 1.7 day versus 2.6 days, *p* < 0.0001) (Table 3). UCLA scores and VAS scores were better in Group 2 than in Group 1 (UCLA *p* = 0.0006, VAS Regular *p* = 0.005, VAS Worst *p* < 0.0001); however HHS and combined ROM were unchanged (Table 4).

**Table 3:** Surgical data

Variable	Group 1	Group 2	*P*-value
Length of Incision (in)	4.5 ± 2.63	4.2 ± 0.49	0.0866
Operation Time (min)	114 ± 18.54	95 ± 14.17	<0.0001^a^
Estimated Blood Loss (mL)	257 ± 100.07	142 ± 76.60	<0.0001^a^
Hospital Stay (days)	2.6 ± 1.02	1.7 ± 0.75	<0.0001^a^

^a^Statistically significant

**Table 4:** Clinical outcomes

Variable	Group 1	Group 2	*P*-value
*Preoperative*			
HHS Score^a^	53 ± 12.44	57 ± 14.91	0.0115*
*Postoperative*			
HHS Score	98 ± 4.10	98 ± 5.49	1.000
UCLA Score	6.3 ± 1.24	7.0 ± 2.05	0.0006*
VAS^b^ Pain: Regular	0.5 ± 0.90	0.2 ± 0.98	0.0050*
VAS Pain: Worst day	2.3 ± 2.34	1.2 ± 2.17	<0.0001*
Combined ROM^c^	289 ± 49.5	291 ± 55.5	0.7376
*Radiographic Data*			
AIA^d^	46 ± 8.03	34 ± 5.36	<0.0001*
Met RAIL Criteria (# Hips, %)	93 (76 %)	239 (99 %)	<0.0001*
Radiolucency (# Hips, %)	2 (1.7 %)	0 (0 %)	0.0444*
Osteolysis (# Hips, %)	0 (0 %)	0 (0 %)	1.000

^a^HHS - Harris Hip Score

^b^VAS – Visual Analog Scale

^c^ROM – Range of Motion

^d^AIA - Acetabular Inclination Angle

*Statistically significant

Radiographic data (Table 4) indicated that acetabular components were placed in a more closed position after implementation of the RAIL guideline (Fig. 1) and the normalized x-ray technique (AIA 35° versus 41° for Group 2 and Group 1, respectively, *p* < 0.0001). Retrospective analysis indicated 28 (24 %) patients in Group 1 had nonideal AIA according to the RAIL criteria; 6 of these patients (25 %) had problematic ion levels and 4 patients (17 %) developed AWRF. After implementation of the RAIL guideline, only 3 patients (1 %) had nonideal AIA and none had AWRF or problematic ion levels. These cases were done in December 2008, January 2009, and March 2011; on follow-up, all of these patients have had normal ion levels and HHS = 100. Since March 2011, no patients have failed to meet RAIL criteria.

Patients in Group 2 had lower mean metal ion levels and were more likely to have normal (*p* = 0.005 for unilateral and *p* = 0.05 for bilateral cases) and optimal ion levels (*p* = 0.0003 for unilateral and *p* = 0.04 for bilateral cases) (Table 5). Cobalt levels in the 242 consecutive high-risk dysplasia patients in Group 2, of whom 70 % were women and 34 % required bearing sizes < 48 mm, were optimal in 99 % of unilateral cases and 88 % of bilateral cases, with no levels > 10 μg/L. All patients with AWRF in Group 1 had ion levels ≥ 15 μg/L. To date, 4 patients in Group 1, but none in Group 2, have developed AWRF (*p* = 0.01) (Table 6).

**Table 5:** Metal ion data

Variables	Group 1 (Pre-2008)			Group 2 (Post-2008)			*P*-values between Group 1 and Group 2	
	Unilateral (*N* = 51)	Bilateral (*N* = 50)	*P*-value	Unilateral (*N* = 118)	Bilateral (*N* = 72)	*P*-value	Unilat I vs II	Bilat I vs II
Co^a^ (μg/L)	1.7 ± 1.90	3.1 ± 2.32	0.0013^b^	1.3 ± 0.93	2.2 ± 1.33	<0.0001^b^	0.0679	0.0076^b^
Cr^a^ (μg/L)	1.6 ± 2.15	2.5 ± 2.04	0.0235^b^	1.2 ± 1.10	1.9 ± 1.67	0.0006^b^	0.1107	0.0775
Follow-Up Date (Yrs)	5.4 ± 1.93	6.5 ± 2.24	0.0095^b^	2.2 ± 1.14	2.5 ± 1.16	0.0821	<0.0001^b^	<0.0001^b^
#, % Patients Tested	105 (87 %)		--	187 (77 %)		--	0.0316^b^	
#, % Levels Converted	17 (33 %)	21 (42 %)	0.3320	26 (22 %)	19 (25 %)	0.5961	0.1416	0.0510
#, % Revised (Excluded)	15 (12 %)	6 (5 %)	0.0394^b^	2 (0.8 %)	0 (0 %)	0.1556	<0.0001^b^	0.0005^b^
#, % Revised AWRF (Excluded)	2 (1.7 %)	2 (1.7 %)	1.000	0 (0 %)	0 (0 %)	1.000	0.0444^b^	0.0444^b^
Normal (#, %)	32 (56 %)	6 (12.5 %)	<0.0001^b^	86 (77 %)	20 (28 %)	<0.0001^b^	0.0058^b^	0.0466^b^
Optimal (#, %)	49 (86 %)	35 (73 %)	0.0949	111 (99 %)	63 (88 %)	0.0007^b^	0.0003^b^	0.0434^b^
Acceptable (#,%)	7 (12.3 %)	12 (25 %)	0.0910	3 (2.7 %)	9 (12.5 %)	0.0085^b^	0.0124^b^	0.0767
Problematic (#, %)	1 (1.8 %)	1 (2.1 %)	0.9045	0 (0 %)	0 (0 %)	1.000	0.1585	0.2187
Potentially Toxic (#, %)	0 (0 %)	0 (0 %)	1.000	0 (0 %)	0 (0 %)	1.000	1.000	1.000

^a^Co – Cobalt, Cr - Chromium

^b^Statistically significant

**Table 6:** Failures requiring revision

Type	Group 1	Group 2	*P*-value
# Cases	121	242	--
*Acetabular Failures*			
Adverse Wear	4 (3.3 %)	0 (0 %)	0.0045*
Loose Acetabular Component	1 (0.8 %)	0 (0 %)	0.1556
Failure of Acetabular Ingrowth	6 (5.0 %)	0 (0 %)	0.0005*
Acetabular Component Shift	0 (0 %)	1 (0.4 %)	0.4777
*Femoral Failures*			
Femoral Neck Fracture	1 (0.8 %)	1 (0.4 %)	0.6171
Femoral Head Collapse	2 (1.7 %)	0 (0 %)	0.0444*
Loose Femoral Component	1 (0.8 %)	0 (0 %)	0.1556
*Other Failures*			
Unexplained Pain	1 (0.8 %)	0 (0 %)	0.1556
*TOTAL FAILURES*	*16 (13.2 %)*	*2 (0.8 %)*	<0.0001*

astericks indicate statistical significance

**Fig. 5:**
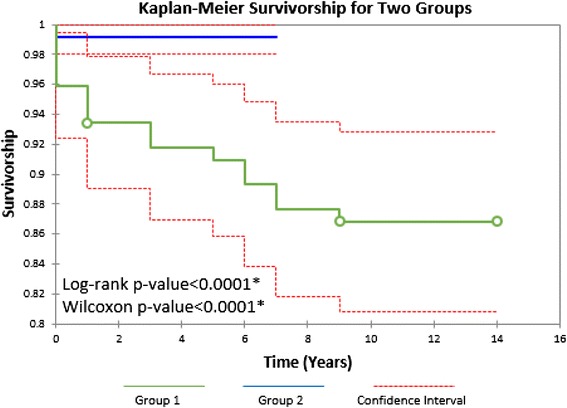
Kaplan-Meier Implant Survivorship Curve for Two Study Groups. Kaplan-Meier survivorship curves for pre-2008 (Group 1) and post-2008 (Group 2) resurfacing procedures on dysplasia patients. Revision of any component was used as endpoint. Plus signs represent censored deaths unrelated to the patient’s hip surgery. (*statistically significant)

**Fig. 6:**
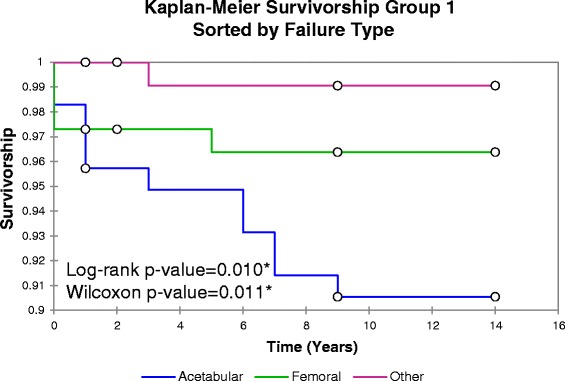
Kaplan-Meier Implant Survivorship Curves, Sorted by Failure Type. Kaplan-Meier survivorship curves for pre-2008 (Group 1) resurfacing procedures on dysplasia patients, categorized by failure type. Markers at the bottom of a step represent failure while markers at the top of steps represent censored deaths unrelated to the patient’s hip surgery

Patients in Group 2, by definition, have had shorter durations of follow-up than those in Group 1. However, all patients in Group 2 have had at least 2 years of follow-up, and some have had 7 years of follow-up. At 2 years, there were 8 implant failures in Group 1 versus 2 in Group 2 (6.6 % vs. 0.8 %, *p* = 0.002). Seven-year Kaplan-Meier implant survivorship was higher in Group 2 (99 % vs. 89 %, *p* < 0.0001) (Figs. 5 and 6). Failures due to FAI or head collapse have not occurred in Group 2 (Tables 6 and 7). Complications and reoperations are reported in Table 8.

**Table 7:** Failures Occurring before 2 Years Postoperatively

Type	Group 1	Group 2	*P*-value
# Cases	121	242	--
*Acetabular Failures*			
Failure of Acetabular Ingrowth	5 (4.1 %)	0 (0 %)	0.0015*
Acetabular Component Shift	0 (0 %)	1 (0.4 %)	0.4777
*Femoral Failures*			
Femoral Neck Fracture	1 (0.8 %)	1 (0.4 %)	0.6171
Femoral Head Collapse	2 (1.7 %)	0 (0 %)	0.0444*
*TOTAL FAILURES*	*8 (6.6 %)*	*2 (0.8 %)*	0.0015*

astericks indicate statistical significance

**Table 8:** Complications and reoperations

Type	Group 1	Group 2	*P*-value
# Cases	121	242	--
*Complications*			
* Acetabular Complications*			
Acetabular Component Shift	1 (0.8 %)	1 (0.4 %)	0.6171
* Other Complications*			
Psoas Tendonitis	1 (0.8 %)	0 (0 %)	0.1556
Hip Dislocation	1 (0.8 %)	1 (0.4 %)	0.6171
Abductor Tear	1 (0.8 %)	0 (0 %)	0.1556
Deep Vein Thrombosis	0 (0 %)	1 (0.4 %)	0.4777
*TOTAL COMPLICATIONS*	4 (3.3 %)	3 (1.2 %)	0.1770
* Reoperations*			
Femoral Neck Fracture	1 (0.8 %)	0 (0 %)	0.1556
Late IT Fracture	0 (0 %)	1 (0.4 %)	0.4777
Fascial Healing Defect	0 (0 %)	1 (0.5 %)	0.4777
*TOTAL REOPERATIONS*	1 (0.8 %)	2 (0.8 %)	1.000

One patient in each group had a single dislocation from extreme movements; these patients did not require reoperation or revision for hip instability. Seven patients in Group 1 and six in Group 2 have had 3D studies to evaluate residual pain and none have indicated AWRF.

Only two patients in Group 2 (0.8 %) have thus far required revision. A 60-year-old woman with a BMI of 24 and a femoral neck T-score of −0.5 had no intraoperative complications but suffered a femoral neck fracture 4 days postoperatively and was converted to a large metal bearing THA without further problems. A 62-year-old woman with an AIA = 34° on standing pelvis film on postoperative day one was recovering well but was found to have an AIA = 17° on her 6-week follow-up standing AP pelvis x-ray. The well-fixed but malpositioned cup was revised to a Magnum™ Tri-Spike of the same size with subsequent excellent outcome.

## Discussion

The 363 consecutive implants for mild developmental hip dysplasia reported here, performed by a single surgeon with a 96 % follow-up, represent to our knowledge the largest reported series on operative treatment for this condition. The usual limitations of sequential cohort design apply to this study. Since the two groups (cases done before and after mid-2008) were not concurrent, gradual improvements in proficiency in both operative and perioperative techniques could have come into play. Lengths of follow-up were, by definition, shorter in Group 2 (cases done after mid-2008) than in Group 1, although we note that all patients from either group had at least 2 years of follow-up. We identified the most common failure modes for HRA (FAI, AWRF, and EFF) and addressed these causes through a series of interventions: improved acetabular component fixation, improved acetabular component alignment through use of the RAIL guideline based on normalized-to-standing intraoperative x-rays, a bone protection program based on each patient’s bone density and BMI, and uncemented femoral fixation. Patients who underwent HRA for developmental dysplasia after these interventions were in place showed improved two-year failure rates, improved projected 7-year Kaplan-Meier implant survivorships, lower blood ion levels, and better functional results as assessed by UCLA and VAS scores (despite unchanged HHSs).

The heterogeneity of the previously-reported case series of implant surgery for developmental dysplasia complicates comparison of these data with previously-reported studies, most of which focused mainly on grade 4 dysplasia or contained mixtures of all grades. Few studies have limited cases to dysplasia grade 1 and 2, which are amendable to resurfacing. Millis demonstrated a 74 % 10-year success rate with the Bernese osteotomy presumably for low-grade dysplasia [32]. Reports on cemented THA indicate 73 % to 98 % 5- to 10-year survivorships [33, 34]; reports on HRA indicate 92 % to 98 % 5- to 10-year survivorships [34, 35]. We are unaware of mid-term data for uncemented THA in mild dysplasia. Implant survivorship in our 242 patients who had surgery after interventions were all in place compares favorably with previous studies for both THA and HRA (Table 9).

**Table 9:** Literature review

Study	Procedure	Prosthesis	Date range	Diagnosis	Patient **c**ohort		Avg FU (Yrs)	Survivorship	
					Hips	Female		FU	Rate
Amstutz *et al.*	HRA	Conserve Plus	1996–2006	Dysplasia	103	78 %	4.8	8	92 %
McBryde *et al.*	HRA	Birmingham	1997–2004	Dysplasia	96	81 %	4.4	5	97 %
Naal *et al.*	HRA	Birmingham	2002–2005	Dysplasia	32	56 %	3.6	5	94 %
Pagnano *et al.*	THA	Charnley THA	1969–1980	Dysplasia Crowe 2	145	82 %	14	7 12	73 % 56 %
Numair *et al.*	THA	Charnley THA	1965–1987	Dysplasia Crowe 1–3	136	63 %	--	10	98 %
Linde *et al.*	THA	Charnley THA	--	Dysplasia	129	--	--	5 10	93 % 89 %
Millis *et al.*	Bernese Osteotomy	--	1991–1998	Dysplasia	135	86 %	9	5 10	96 % 84 %
Adelani *et al.*	Revision THA	-Varies-	1996–2006	<55 years old	103	66 %	6.7	6.7	69 %
Current Study Group 1	HRA	Corin Hybrid, Biomet ReCap™ Hybrid	2001–2008	Dysplasia	121	71 %	6.4	7 12	90 % 86 %
Current Study Group 2	HRA	Biomet ReCap™ Uncemented	2008–2013	Dysplasia	242	74 %	2.6	7	99 %

Literature comparison of our Group 1 and Group 2 results with results from Amstutz [45], McBryde [46], Naal [47], Pagnano [48], Numair [33], Linde [34], Millis [49], and Adelani [50]

### Acetabular component fixation

FAI, the most common failure mode of arthroplasty for hip dysplasia, occurred mainly in patients with more severely-elongated sockets. Prior to 2007, inadequate supplemental fixation was the usual cause of FAI in cases where very ovoid or flattened sockets could not be made hemispherical [20]. In 2007, Biomet released a socket with supplemental fixation (Magnum™ Tri-Spike) that could be employed for resurfacing. We subsequently began using this implant in all cases where > 30 % of implant uncoverage was judged to exist. Selectively using this implant in only 5 % of severe cases allowed us to eliminate FAI as a failure mode.

### Acetabular component alignment

AWRF, the second-most-common failure mode of implant surgery for dysplasia, results largely from suboptimal implant design and alignment. Some authors have suggested (without evidence) that this failure mode is a random event related to metal allergy [36]. DeSmet [22] demonstrated that *inadequate functional coverage arc* of the implanted acetabular component is the underlying cause of excessive metal wear; overwhelming data now confirms his concepts [37–39]. Inadequate functional coverage, which results in edge loading, loss of fluid film lubrication, and an extremely high wear rate, is caused by suboptimal implant design and unsatisfactory surgeon placement. Inadequate functional coverage and suboptimal alignment lead to excessive metal debris deposition in tissues, causing an inflammatory reaction, which we have called AWRF. The logical conclusion from DeSmet’s work is that AWRF can be avoided in three ways: select against patients requiring smaller bearing sizes (mostly women), redesign acetabular components to increase coverage arc across all sizes (Wright Conserve Biofoam component), or learn to consistently implant smaller sizes more horizontally to avoid edge loading [23]. Herein, we demonstrate the efficacy of the third option.

The DePuy ASR had a lower coverage arc than all other designs and was therefore very prone to AWRF. All current designs (except the new Conserve Biofoam 170°) have coverage arcs that increase with bearing size (e.g. Biomet Magnum™ varies from 40-mm bearing with 156°, to 60-mm bearing with 164°). Therefore, smaller bearing sizes in all brands are more prone to AWRF, leading to females being more susceptible to this mode of failure (women require a mean bearing size of 46 mm while men need a mean of 52 mm, in our experience).

We confirm DeSmet’s hypothesis [22] by demonstrating that implants placed by RAIL criteria (a safe zone for implanting HRA components based on bearing size) avoid high ion levels [23] (Fig. 1). To sum, smaller sizes necessitate a more horizontal position. No metal ion levels > 10 μg/L or instances of AWRF occurred when component positions met our RAIL. By mid-2008, we established intraoperative x-ray techniques [40] to reliably place acetabular components within the RAIL guidelines, and to date have observed neither edge-loading nor AWRF in this high-risk population. In a previous study [39], we had no recurrences of AWRF after revision. Our implant survival for 58 HRA revisions (mean patient age = 50 years) was 97 % at a mean of 5 years. Fear of the consequences of AWRF has been the primary reason that the use of resurfacing has declined. In this study, we have shown that AWRF is completely preventable by proper acetabular component positioning, and previously, we have shown that revision surgery, when done correctly, results in excellent outcomes.

### Bone protection program and uncemented fixation

EFF, the third principal failure mode of implants in dysplasia patients, correlates with low bone density and high BMI, as we demonstrated by multivariate analysis previously [26]. At the same time, we showed that a bone management program consisting of restricted weight-bearing and alendronate could eliminate these failures in high-risk patients. We subsequently expanded this protocol to include intermediate-risk patients. Additionally, we began using an uncemented Biomet ReCap™ femoral component for resurfacing in 2007 with hopes of reducing femoral failure modes. Since 2008, they have been used exclusively. While the early outcomes have been excellent [4], and the failure rate in osteonecrosis (ON) cases has been reduced [41], we have not previously noticed a lower EFF rate with the uncemented femoral component [26]. In this study, the EFF rate was reduced for Group 2, but based on these results, we cannot attribute this improvement to one particular intervention. Based on our previous studies, we demonstrated that EFF reduction was primarily due to our bone management program rather than the uncemented femoral component.

We are able to show unequivocally that results have improved; we are also able to show that certain failure modes are now less prevalent. But because numerous changes were made, we cannot with certainty understand the exact interplay between variables. There was no single, clear transition date where all improvements were simultaneously initiated; rather, they were gradually developed and instituted in a continual improvement process. Future studies using regression analyses may be helpful in determining relationships. Next, we currently lack substantial data on patients with advanced dysplasia, but these results provide excellent insight into mild cases of dysplasia. We also note the current limitation of external validity due to the nature of the single-surgeon study design. Direct comparison of the two groups was difficult because they were not concurrent; however, all patients had a minimum of 2 years follow-up; therefore, comparison of failures by 2 years was valid.

The strengths of this study far outweigh the limitations. This investigation was based on detailed, prospectively collected data on consecutive patients. The follow-up rate was 96 %. Failures that were revised elsewhere were also recorded. Major changes in our technique were carefully documented in our database. Lastly, the demographics of the patients had minimal variation over time, allowing for more direct comparisons.

## Conclusions

A substantial body of literature suggests that HRA, compared with THA, provides favorable outcomes in such areas as hip stability, gait, and activity tolerance [3, 4, 8–13, 42, 43]. However, HRA remains a technically-demanding procedure, is seldom taught in residency programs, and is stigmatized by concerns that include metal ion levels and implant survivorship. Published tabulations of registry data indicate that some surgeons have difficulty achieving implant survivorship results comparable to those of stemmed THA procedures [44]. However, a growing body of literature now indicates that surgeons experienced at resurfacing can achieve favorable implant survivorship, especially in men [5, 35, 45]. We have extended this observation to include women, specifically those with developmental dysplasia. It is our expectation that surgeons striving for expertise at HRA will incorporate the interventions described here in order to offer the functional advantages of HRA to additional patient populations. We await the experiences of others as well as the longer-term follow-up results on our own patients with great interest.

We conclude that:

- With sufficient experience, hip resurfacing arthroplasty (HRA) can be performed with a success rate that compares favorably to that of standard stemmed total hip arthroplasty in dysplasia patients (99 % 7-year survivorship).
- Dislocations (0.5 %) and revisions for instability (0 %) are rare when HRA is used in high-risk (for instability) dysplasia patients.
- Adverse wear related failures (AWRF) can be completely eliminated, and optimal ion levels can routinely be achieved in high-risk (for AWRF) dysplasia patients if the Relative Acetabular Inclination Limit (RAIL) is observed.
- The RAIL can be achieved in a high percentage of cases (99 %) when a normalized intraoperative x-ray technique is used.
- Failure of acetabular component fixation can be avoided (0 %) if an acetabular component with a supplemental fixation option is selectively (5 %) employed.
- Early femoral failures can be reduced when a comprehensive bone management protocol is followed.
- Uncemented femoral components are highly successful, but we do not have the data to show if the lower rate of femoral complications observed can be assigned to them.

## Abbreviations

AI, anterior inferior; AIA, acetabular inclination angle; AP, anterior posterior; AS, anterior superior; AWRF, adverse wear related failure; BMI, body mass index; DEXA, dual energy x-ray absorptiometry; EFF, early femoral failure (fracture or head collapse before 1 year); FAI, failure of acetabular ingrowth; HHS, Harris hip score; HRA, hip resurfacing arthroplasty; OR, operating room; RAIL, relative acetabular inclination limit; ROM, range-of-motion; TAL, transverse acetabular ligament; THA, total hip arthroplasty; UCLA, University of California at Los Angeles; VAS, visual analog scale; WBAT, weight-bearing as tolerated
